# Assessment of a Large Language Model’s Responses to Questions and Cases About Glaucoma and Retina Management

**DOI:** 10.1001/jamaophthalmol.2023.6917

**Published:** 2024-02-22

**Authors:** Andy S. Huang, Kyle Hirabayashi, Laura Barna, Deep Parikh, Louis R. Pasquale

**Affiliations:** 1 Department of Ophthalmology, Icahn School of Medicine at Mount Sinai, New York, New York; 2 Department of Ophthalmology, Massachusetts Eye and Ear, Harvard Medical School, Boston

## Abstract

**Importance:**

Large language models (LLMs) are revolutionizing medical diagnosis and treatment, offering unprecedented accuracy and ease surpassing conventional search engines. Their integration into medical assistance programs will become pivotal for ophthalmologists as an adjunct for practicing evidence-based medicine. Therefore, the diagnostic and treatment accuracy of LLM-generated responses compared with fellowship-trained ophthalmologists can help assess their accuracy and validate their potential utility in ophthalmic subspecialties.

**Objective:**

To compare the diagnostic accuracy and comprehensiveness of responses from an LLM chatbot with those of fellowship-trained glaucoma and retina specialists on ophthalmological questions and real patient case management.

**Design, Setting, and Participants:**

This comparative cross-sectional study recruited 15 participants aged 31 to 67 years, including 12 attending physicians and 3 senior trainees, from eye clinics affiliated with the Department of Ophthalmology at Icahn School of Medicine at Mount Sinai, New York, New York. Glaucoma and retina questions (10 of each type) were randomly selected from the American Academy of Ophthalmology’s commonly asked questions Ask an Ophthalmologist. Deidentified glaucoma and retinal cases (10 of each type) were randomly selected from ophthalmology patients seen at Icahn School of Medicine at Mount Sinai–affiliated clinics. The LLM used was GPT-4 (version dated May 12, 2023). Data were collected from June to August 2023.

**Main Outcomes and Measures:**

Responses were assessed via a Likert scale for medical accuracy and completeness. Statistical analysis involved the Mann-Whitney *U* test and the Kruskal-Wallis test, followed by pairwise comparison.

**Results:**

The combined question-case mean rank for accuracy was 506.2 for the LLM chatbot and 403.4 for glaucoma specialists (n = 831; Mann-Whitney *U* = 27976.5; *P* < .001), and the mean rank for completeness was 528.3 and 398.7, respectively (n = 828; Mann-Whitney *U* = 25218.5; *P* < .001). The mean rank for accuracy was 235.3 for the LLM chatbot and 216.1 for retina specialists (n = 440; Mann-Whitney *U* = 15518.0; *P* = .17), and the mean rank for completeness was 258.3 and 208.7, respectively (n = 439; Mann-Whitney *U* = 13123.5; *P* = .005). The Dunn test revealed a significant difference between all pairwise comparisons, except specialist vs trainee in rating chatbot completeness. The overall pairwise comparisons showed that both trainees and specialists rated the chatbot’s accuracy and completeness more favorably than those of their specialist counterparts, with specialists noting a significant difference in the chatbot’s accuracy (*z* = 3.23; *P* = .007) and completeness (*z* = 5.86; *P* < .001).

**Conclusions and Relevance:**

This study accentuates the comparative proficiency of LLM chatbots in diagnostic accuracy and completeness compared with fellowship-trained ophthalmologists in various clinical scenarios. The LLM chatbot outperformed glaucoma specialists and matched retina specialists in diagnostic and treatment accuracy, substantiating its role as a promising diagnostic adjunct in ophthalmology.

## Key Points

**Question:**

Can a large language model (LLM) chatbot provide accurate and complete responses compared with fellowship-trained ophthalmologists in managing glaucoma and retina diseases?

**Findings:**

In this cross-sectional study, with responses graded using a Likert scale, the LLM chatbot demonstrated comparative proficiency, largely matching if not outperforming glaucoma and retina subspecialists in addressing ophthalmological questions and patient case management.

**Meaning:**

The findings underscore the potential utility of LLMs as valuable diagnostic adjuncts in ophthalmology, particularly in highly specialized and surgical subspecialties of glaucoma and retina.

## Teaser

This cross-sectional study compares the diagnostic accuracy and comprehensiveness of responses from a large language model chatbot with those of fellowship-trained glaucoma and retina specialists on ophthalmological questions and real patient case management.

## Introduction

Large language models (LLMs) are increasingly being integrated into medical decision-making and patient education and have the potential to be a medical artificial intelligence catalyst in ophthalmology.^1,2,3,4^ LLM chatbots have demonstrated encouraging and consistent performances on stimulated Ophthalmic Knowledge Assessment Program examination questions.^3^ Furthermore, the diagnostic capabilities of LLM chatbots compared with 3 ophthalmology trainees for glaucoma and 2 specialists for retina indicate their potential role in enhancing objective and efficient clinical diagnoses.^1,2,5^

While these studies showcase the potential of LLM chatbots in specific domains, a broader evaluation of their accuracy, including in comparison with attending-level ophthalmologists, is warranted, particularly for addressing real-life clinical case scenarios.^2,5,6,7^ In this study, we compared an LLM chatbot’s responses with those of fellowship-trained glaucoma and retina specialists to explore the potential of LLMs in clinical ophthalmology.

## Methods

### Study Design and Participants

This was a comparative, single-center, cross-sectional study adhering to the Strengthening the Reporting of Observational Studies in Epidemiology (STROBE) reporting guideline. The 13 participants subsequently rated the responses in a masked fashion, amounting to a dataset of 1271 ratings and 1267 ratings for accuracy and completeness, respectively (eFigure 1 in Supplement 1). The Mount Sinai Institutional Review Board approved the study, which involved 15 participants, comprising 12 board-certified, fellowship-trained subspecialists (8 in glaucoma and 4 in retina) and 3 ophthalmology trainees (2 fellows and a senior resident). The mean (SD) and median (IQR) practice duration were 11.7 (13.5) years and 6 (19.5) years, respectively. All participants provided written informed consent.

### Question and Case Selection

Glaucoma and retina questions (10 of each type of commonly asked questions by patients) were randomly selected from the Ask an Ophthalmologist section of the American Academy of Ophthalmology website. Permission was obtained from the American Academy of Ophthalmology to use this content. Deidentified glaucoma and retinal cases (10 of each type) were randomly selected from ophthalmology patients seen at Icahn School of Medicine at Mount Sinai–affiliated clinics. For case selection, before random selection, we curated a pool of cases to be balanced in terms of diversity and complexity. See the eAppendix in Supplement 1 for the clinical cases we used.

### LLM Chatbot Prompting

We used GPT-4 (OpenAI), an advanced LLM that was initially introduced in 2022. A single investigator (A. S. H.) prompted GPT-4 (version dated May 12, 2023) for all queries. Its role was defined as a medical assistant, delivering concise answers to emulate an ophthalmologist’s response (eFigure 2 in Supplement 1). Case-centered inquiries demanded a clear assessment and plan, reflecting the format for medical record documentation. Instructions were provided to openly use medical abbreviations, bereft of any explanations, to ensure the chatbot’s responses mimicked the style of ophthalmology notes.

### Likert Scale Definitions

Answer accuracy was measured on a 10-point Likert scale. Scores between 1 and 2 represented very poor or unacceptable inaccuracies; 3 and 4, poor accuracy with potentially harmful mistakes; 5 and 6, moderate inaccuracies that could be misinterpreted; 7 and 8, good quality with only minor, nonharmful inaccuracies; and 9 and 10, very good accuracy that was devoid of any inaccuracies. Medical completeness was assessed on a 6-point scale. Scores of 1 to 2 indicated that the response was incomplete and missed significant parts of the question or management; 3 to 4, the response was adequate in providing the basic necessary information; and 5 to 6, the answer was medically comprehensive, delving into broad context and offering additional pertinent and nuanced details.

### Objective and End Points

We compared answers to clinical questions and case management generated by GPT-4 and fellowship-trained retina and glaucoma specialists. We compared the accuracy and completeness of answers, evaluated using a Likert scale, which aligns with a validated approach.^6^ Secondary end points explored rating differences between trainees and attendings to assess whether the level of training influenced the perception of the LLM’s responses.

### Measures to Minimize Bias

Participants also rated the responses but were masked to the origin of the other replies, and scores for their responses were censored. We also used randomization in the response order to reduce bias. Specialists were expressly instructed against harnessing LLMs to craft their answers. Both specialists and the LLM chatbot were instructed to respond in a consistently structured bullet-point format for clarity and coherence.

### Statistical Analysis

Descriptive statistics—primarily medians, mean ranks, and quartiles—were computed for responses. Due to the ordinal nature of Likert scale data and the nonnormal distribution of the data, nonparametric tests, specifically the Mann-Whitney *U* test and the Kruskal-Wallis test, were used. The level of significance was set at *P* < .05, and all tests were 2-tailed. The Mann-Whitney *U* test was used to determine differences in accuracy and completeness between the chatbot and the glaucoma or retina specialists. The Kruskal-Wallis test identified global differences between the chatbot, specialists, and trainees, followed by Dunn’s post-hoc pairwise comparison. We used SPSS version 29.0.1.0 (IBM) for all analyses.

## Results

The combined question-case mean rank for accuracy was 506.2 for the LLM chatbot and 403.4 for glaucoma specialists (n = 831; Mann-Whitney *U* = 27976.5; *P* < .001), and the mean rank for completeness was 528.3 and 398.7, respectively (n = 828; Mann-Whitney *U* = 25218.5; *P* < .001) (Table 1; eFigure 3A in Supplement 1). The mean rank for accuracy was 235.3 for the LLM chatbot and 216.1 for retina specialists (n = 440; Mann-Whitney *U* = 15518.0; *P* = .17), and the mean rank for completeness was 258.3 and 208.7, respectively (n = 439; Mann-Whitney *U* = 13123.5; *P* = .005) (Table 1; eFigure 3B in Supplement 1). Differences existed between specialists and trainees in both accuracy Likert scoring (n = 1271; Kruskal-Wallis *H* = 44.36; *P* < .001) and completeness Likert scoring (n = 1268; Kruskal-Wallis *H* = 88.27; *P* < .001). The Dunn test revealed a difference between all pairs, except specialist vs trainee in rating chatbot completeness (Figure; Table 2). However, the overall pairwise comparisons showed that both trainees and specialists rated the chatbot’s accuracy and completeness more favorably than those of their specialist counterparts, with specialists noting a significant difference in the chatbot’s accuracy (*z* = 3.23; *P* = .007) and completeness (*z* = 5.86; *P* < .001) (Figure).

**Table 1.** Comparison of Large Language Model (LLM) Chatbot vs Ophthalmology Specialists on Accuracy and Completeness in Glaucoma and Retina Questions and Cases

Measure	Total, No.	Mann-Whitney *U* statistic	*P* value	Mean rank	
				LLM chatbot	Specialist
**Glaucoma**					
Question accuracy	450	6572.5	<.001	303.5	214.6
Case accuracy	381	7549.0	.67	197.4	190.1
Combined accuracy	831	27 976.5	<.001	506.2	403.4
Question completeness	449	5711.0	<.001	318.2	212.0
Case completeness	379	7122.5	.32	204.5	188.0
Combined completeness	828	25 218.5	<.001	528.3	398.7
**Retina**					
Question accuracy	220	3403.0	.03	127.4	105.5
Case accuracy	220	4396.5	.70	107.6	111.4
Combined accuracy	440	15 518.0	.17	235.3	216.1
Question completeness	220	2828.5	.008	138.9	102.1
Case completeness	219	3774.5	.22	119.0	107.3
Combined completeness	439	13 123.5	.005	258.3	208.7

**Figure.**
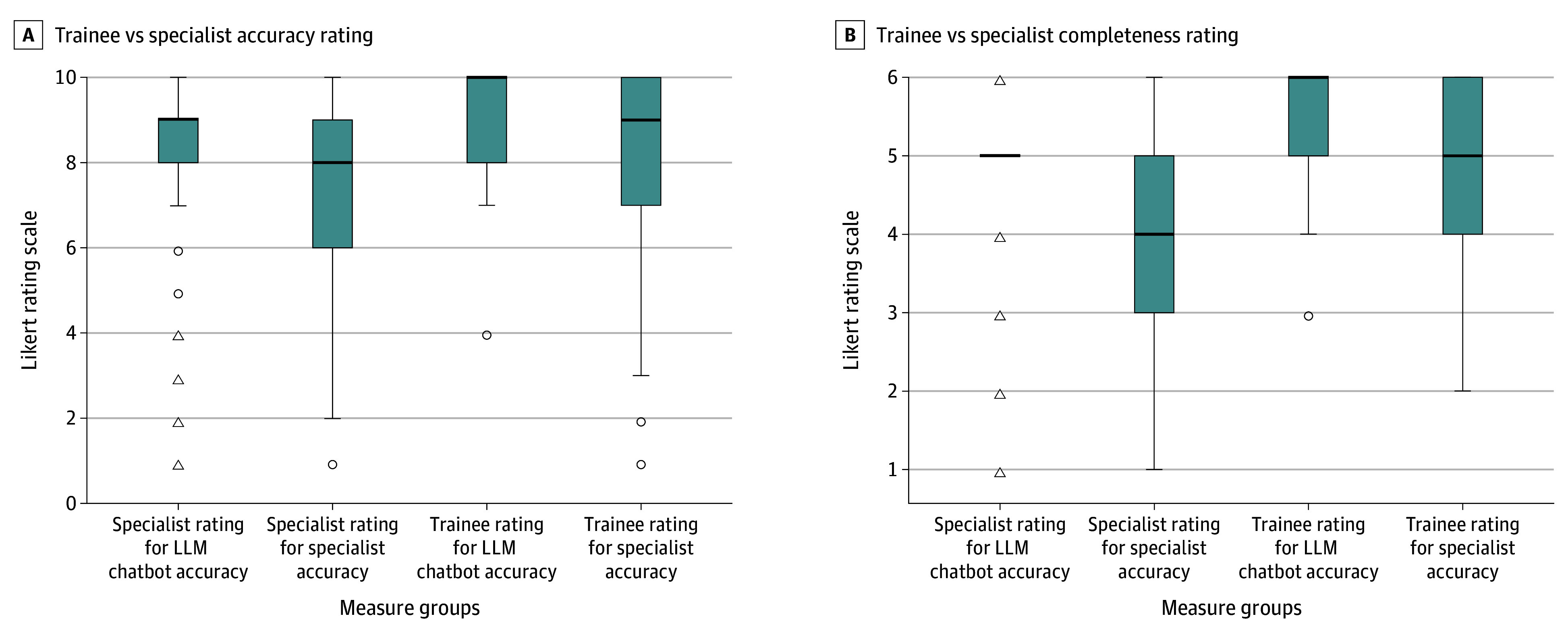
Comparative Trainee and Specialist Ratings on Accuracy and Completeness of Large Language Model (LLM) Chatbot and Specialist Responses in Glaucoma and Retina Clinical Scenarios In the presented box plots, the box indicates the IQR the between first and third quartile; the center line indicates the median of the dataset; the whiskers indicate 1.5-fold the IQR; circles indicate mild outliers (values between 1.5-fold to 3-fold the IQR); and triangles indicate extreme outliers (more than 3-fold the IQR). See Table 2 for pairwise comparison of the 4 different groups.

**Table 2.** Comparison of Trainee vs Specialist Ratings for Large Language Model (LLM) Chatbot and Specialist Accuracy and Completeness^a^

Comparison	Test statistic	SE	Standard test statistic	Adjusted *P* value^b^
**Accuracy rating**				
Trainee vs specialist in rating specialist	75.09	24.30	3.09	.01
Trainee vs specialist in rating LLM chatbot	179.51	55.84	3.22	.008
Trainee rating: LLM chatbot vs specialist	210.81	51.24	4.11	.001
Specialist rating: LLM chatbot vs specialist	106.38	32.90	3.23	.007
**Completeness rating**				
Trainee vs specialist in rating specialist	137.02	23.99	5.71	<.001
Trainee vs specialist in rating LLM chatbot	140.91	55.04	2.56	.06
Trainee rating: LLM chatbot vs specialist	193.87	50.53	3.84	.001
Specialist rating: LLM chatbot vs specialist	189.97	32.44	5.86	<.001

^a^ Due to the limited number of trainee participants (n = 3), there may be constraints on the generalizability of these findings.

^b^ Significant values have been adjusted by the Bonferroni correction for multiple tests.

## Discussion

The LLM chatbot’s performance demonstrated superiority in glaucoma diagnosis and treatment compared with fellowship-trained specialists. The chatbot’s performance relative to retina specialists showed a more balanced outcome, matching them in accuracy but exceeding them in completeness. The LLM chatbot exhibited consistent performance across pairwise comparisons, maintaining its accuracy and comprehensiveness standards for the questions and clinical scenarios. The enhanced performance of the chatbot in our study compared with other evaluations could be attributed to the refined prompting techniques used (eFigure 1 in Supplement 1), specifically instructing the model to respond as a clinician in an ophthalmology note format.

Recent research aligns with our findings. Delsoz et al^1^ reported the diagnostic proficiency of an LLM chatbot in glaucoma as comparable with ophthalmology residents. Rojas-Carabali et al^8^ reported the performance of a chatbot in uveitis diagnosis to be slightly behind uveitis-trained ophthalmologists but consistent in management plans. Investigating rare eye disease, Hu et al^9^ highlighted an LLM chatbot’s potential as a support tool, especially for junior ophthalmologists. Another corneal disease study emphasized the updated chatbot’s superiority over its predecessor and its promising accuracy, although not consistently surpassing human experts.^10^ Another study found that LLM-generated ophthalmic advice to online forum questions is nearly as safe and accurate as ophthalmologists.^4^ These studies emphasize the emerging role of LLM chatbots in ophthalmology, highlighting their strengths and areas needing refinement. While prior studies test the factual clinical knowledge of various LLMs, this work shows that an LLM chatbot can synthesize clinical data and report an impression and plan comparable with seasoned subspecialists.

### Limitations

This study has limitations. This single-center, cross-sectional study only evaluated LLM proficiency at a single time point among 1 group of attendings and trainees. A longitudinal, multicentered evaluation on a larger dataset would offer additional insights into the consistency and adaptability of future LLMs. Our findings, while promising, should not be interpreted as endorsing direct clinical application due to chatbots’ unclear limitations in complex decision-making, alongside necessary ethical, regulatory, and validation considerations not covered in this report.

## Conclusions

In this study, an LLM chatbot had comparative diagnostic accuracy and completeness in glaucoma and retina against fellowship-trained ophthalmologists in both clinical questions and clinical cases. These findings support the possibility that artificial intelligence tools could play a pivotal role as both diagnostic and therapeutic adjuncts.

## Notes

Supplement 1. **eFigure 1.** Flowchart of Study Design for Evaluation of GPT-4 and Subspecialty Attending Responses in Clinical Ophthalmology Scenarios **eFigure 2.** Comparison of GPT-4–Generated Answers With Fellowship-Trained Glaucoma Ophthalmologists **eFigure 3.** GPT vs Specialist in Glaucoma and Retina Clinical Scenarios **eAppendix.** Glaucoma and Retina Ophthalmology Patient Cases

Supplement 2. Data Sharing Statement
