# Supplementary material for: Assessment of a Large Language Model’s Responses to Questions and Cases About Glaucoma and Retina Management
Source: JAMA Ophthalmol. 2024 Feb 22;142(4):371–5. doi: 10.1001/jamaophthalmol.2023.6917 (PMC10884943; doi:10.1001/jamaophthalmol.2023.6917)
Supplement: Supplement 1. — eFigure 1. Flowchart of Study Design for Evaluation of GPT-4 and Subspecialty Attending Responses in Clinical Ophthalmology Scenarios eFigure 2. Comparison of GPT-4–Generated Answers With Fellowship-Trained Glaucoma Ophthalmologists eFigure 3. GPT vs Specialist in Glaucoma and Retina Clinical Scenarios eAppendix. Glaucoma and Retina Ophthalmology Patient Cases [file jamaophthalmol-e236917-s001.pdf]

Supplemental Online Content

Huang AS, Hirabayashi K, Barna L, Parikh D, Pasquale LR. Assessment of a large language model's responses to questions and cases about glaucoma and retina management. *JAMA Ophthalmol*. Published online February 22, 2024. doi:10.1001/jamaophthalmol.2023.6917

eFigure 1. Flowchart of Study Design for Evaluation of GPT-4 and Subspecialty Attending Responses in Clinical Ophthalmology Scenarios

eFigure 2. Comparison of GPT-4–Generated Answers With Fellowship-Trained Glaucoma Ophthalmologists

eFigure 3. GPT vs Specialist in Glaucoma and Retina Clinical Scenarios

eAppendix. Glaucoma and Retina Ophthalmology Patient Cases

This supplementary material has been provided by the authors to give readers additional information about their work.

© 2024 Huang AS et al. *JAMA Ophthalmology*.

eFigure 1. Flowchart of Study Design for Evaluation of GPT-4 and Subspecialty Attending Responses in Clinical Ophthalmology Scenarios

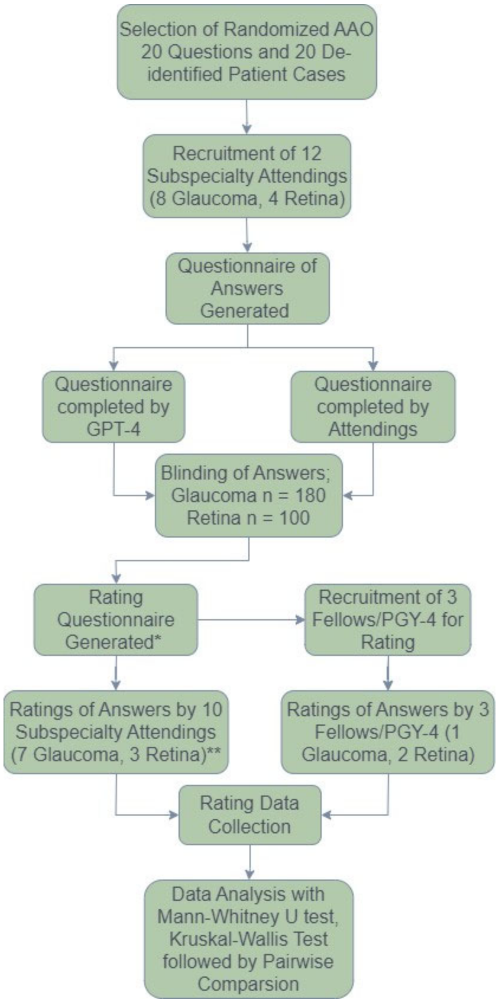

Supplementary Figure 1: This flowchart depicts the methodological framework of the study beginning with the selection of randomized questions (10 glaucoma and 10 retina) and de-identified patient cases (10 glaucoma and 10 retina), followed by the recruitment of subspecialty attending ophthalmologists and the generation of responses. The process continues with GPT-4 and attending subspecialists independently completing questionnaires, blinding of both responses, and subsequent rating by both attending and trainees (1 glaucoma fellow, 1 retina fellow, and 1 PGY-4 resident). The final stages include data collection of ratings and statistical analysis using Mann-Whitney U test, Kruskal-Wallis test, and pairwise comparison of accuracy and completeness of responses.

* Some variability in the dataset arises from instances where raters inadvertently skipped certain items or made obvious typos, and such anomalies were subsequently removed to maintain the integrity of the data. Furthermore, one glaucoma case was excluded from the analysis due to an identified error within the case itself, slightly reducing the total number of responses.

** The questionnaire for rating the accuracy and completeness of answers was divided into two parts for the glaucoma answers due to larger number of answers generated (compared to Retina) and were given the option to complete one or both parts. 5 glaucoma attendings completed half the rating questionnaire, and 2 glaucoma attendings and 1 glaucoma fellow completed both parts of the rating questionnaire. To maintain objectivity in the evaluation process, subspecialist attendings who generated answers were not permitted to rate their responses. For clarity and self-reference, their own responses were highlighted in the questionnaire they received.

NB: Due to the nonparametric nature of our data, 95% confidence intervals were not included. Instead, we have provided interquartile ranges to represent the spread and variability in the data.

© 2024 Huang AS et al. *JAMA Ophthalmology*.

eFigure 2. Comparison of GPT-4–Generated Answers With Fellowship-Trained Glaucoma Ophthalmologists

GPT-4

I am an ophthalmologist, and you are assisting me with medical management and answering questions and cases. Please answer succinctly, accurately, and precisely. It is important to be concise with the answers. Generate the answers similar to an ophthalmologist's response, with variations in complexity in sentences, and do not plagiarize. When appropriate, please use evidence-based medicine for your answers, but answer just the question/case. Complete the assessment and plan as two separate sections in bullet points. All responses should use abbreviations used by ophthalmologists as to not allow readers to distinguish between GPT-4 and a real ophthalmologist. Do you understand?

Yes, I understand. I will provide concise, accurate, and precise medical information in the manner of an ophthalmologist, using appropriate abbreviations and evidence-based medicine where relevant. I will structure the responses with separate sections for assessment and plans in bullet points.

Provide an assessment and plan (step-by-step) based on the history and physical examination. Please provide this via diagnosis (or possible differential diagnosis if unclear) and then provide a next step management and treatment. Be specific with this, but also very high emphasis on being concise. Organize all of this as an ophthalmologist would write in their note in a hospital setting. Both assessments/plans should be bullet points. Under the plan, just include management. Include more abbreviations and brevity to mimic ophthalmologists' notes. Abbreviations do not need to be defined.

Understood. Please provide the specific patient history and physical examination details for the assessment and plan.

One liner: 78 y/o M h/o CACG s/p LI OU, CEIOL OU s/p Kahook goniotomy OS; gtts: Alphagan 2/2, Cosopt 2/2, Xalatan hs/hs, Rhopressa hs/hs.
Exam: VA OD 20/100; OS HM. IOP 7/7; Tmax 23/23. PERRL, no rAPD OU. Slit lamp: LL: normal OU. Conj/sclera: white and quiet. Cornea: PEE OU. AC: deep and quiet OU. Iris: patent LI OU. Lens: PCIOL OU. CCT: 529/527. Gonio: grade 3 OU, posterior trab mesh-scleral spur OU. Dilated fundus exam: Disc: 0.7 OU. Macula: normal, flat OU. Vessels: normal OU. Periphery: normal OU. Perimetry: Inferotemporal islands OU. RNFL: 61/61, thinning OU.

Assessment:

- Chronic Angle Closure Glaucoma (CACG) OU, stable on current treatment, s/p LI OU, s/p Kahook goniotomy OS.
- CEIOL OU.
- Decreased VA OD and OS, cause to be determined.

Plan:

- Continue Alphagan 2/2, Cosopt 2/2, Xalatan hs/hs, Rhopressa hs/hs.
- IOP check q3mo.
- VF and OCT in 6mo for progression.
- Consider additional workup for decreased VA including OCT macula, FFA, and/or consult retina.
- If IOP increases or VF loss progresses, consider revision of Kahook goniotomy or glaucoma drainage implant.
- Annual DFE.

Assessment:
- Advanced/severe CACG L>R with now open angles on max meds post Kahook OS
- Thin CCT, IOP may be higher than measured
- Optic nerves symmetrical cupping
- NFL symmetrical thinning
- Severe VF loss, Reduced VA L>R

Plan:
- Patient is dosing meds 6 times per day per eye, consider simplifying regimen. Substitute Rocklatan for Xal and Rhopressa.
- Review compliance and observe drop instillation by patient/caregiver.
- Continue other gtts.
- NLO and eyelid closure and dosing regimen reviewed.
- RV 3 months HVF.

Assessment:
- severe CACG OU s/p LI and s/p KDB OS
- Pseudophakia OU
- Dry eye syndrome OU

Plan:
- Very severe disease OU as evidence by HVF and OCT RNFL.
- IOP excellent in the single digits with maximum topical drops.
- Encourage drop compliance - counsel on drop spacing and timing to maximize therapeutic effect.
- If concern for drop compliance or intolerance to drops schedule would offer glaucoma surgery as option but given advanced nature and low IOPs it would be best to continue to follow as is.
- If inferotemporal islands on HVF are changing and OCT RNFL continues to thin then would offer surgery as way to reduce drop burden and have better 24-hour IOP control without relying on patient to use drops.
- Recheck for APD next visit.
- Low vision refraction if not recent - monocular precautions - full-time polycarbonate wear.
- RTC 3 months +/- OCT RNFL +/- HVF (depends on how dated the testing is).

Assessment:
- Mixed-mechanism glaucoma, severe OU

Plan:
- Well-controlled, continue same drops.
- F/u 6 months
- Yearly HVF 24-2 OU. If OCT RNFL bottomed out, get OCT GCA instead OU.

Assessment:
- Advanced CACG OU

Plan:
- Continue cos 2/2, Alphagan 2/2, Xal 1/1, Rho 1/1.
- RTC 3 months repeat HVF.

Assessment:
- CACG probably advanced s/p LI, OU; s/p Kahook goniotomy OS. There is discordance between the vision, IOP profile, CDR, and structural/functional tests. It is unclear if IOP was much higher than captured from the current notes or if some of the current exam findings are flatly inaccurate. Currently, IOP is at episcleral venous level using maximum topical medical therapy.
- Poor vision OU that could be from glaucoma but suspicion for another ophthalmic disease should be high.

Plan:
- Continue current glaucoma medicines for now.
- Repeat exam, especially the assessment of the optic nerves to see if cupping is far worse than reported here. Bruchs membrane opening report on OCT can confirm that the retinal nerve fiber layer loss is profound enough to explain the current visual acuity. Nb: The global RNFL thickness readings provided can be consistent with much better Snellen acuity.
- Swept-source OCT of the optic nerve head can resolve the prelaminar optic nerve anatomy and determine if the CDR of 0.7 is misleading and if there is considerable pre-laminar gliosis that could make the CDR seem small.
- Get macula OCT to r/o macula pathology.
- Consider retina consult and/or neuro-ophthalmology consult if decreased vision cannot confidently be attributed to advanced CACG, OU.

Assessment:
- Chronic angle-closure glaucoma OU
Advanced glaucomatous optic neuropathy OU
Extent of visual field loss and visual acuity disproportionate with magnitude and symmetry of optic disc cupping and RNFL loss -- possible rebound of cupping at very low IOP but would expect pallor if this is the case.
IOP well controlled OU with significant reduction from historical maximums, s/p Kahook goniotomy OS and using multiple glaucoma medications OU.
-Pseudophakia OU

Plan:
- Continue current medications OU.
- Obtain old ophthalmic records to assess stability of glaucomatous optic neuropathy OU.
- Given patients age and absence of other symptoms would forego work-up for non-glaucomatous optic neuropathy (no disc pallor mentioned).
- Baseline stereo disc photos (monitor annually).
- If IOP stable without evidence of progression, monitor IOP every 3 months and VF OD and RNFL OU every 6 - 12 months (not possible to obtain reliable automated perimetry OS, so would follow Goldmann, kinetic VF OS or forego VF testing altogether in this eye).
- Obtain baseline VF 24-2 and 10-2 algorithms, using a stimulus size V OD and follow these tests.
- Educate patient about the proper technique for eyedrop instillation, adherence to medication regimen, and compliance with follow up visits.
- Encourage follow-up with internist to monitor HTN.

Assessment:
- CACG s/p LI OU, CEIOL OU s/p Kahook goniotomy OS
- PEE OU
- PCIOL OU

Plan:
- Given IOP on low side and advanced age, change Cosopt and Alphagan to Simb 2/2.
- Recheck IOP 4-6 weeks.
- Preservative free artificial tears as needed.

Assessment:
- Severe stage CACG with open angles OU s/p phaco/IOL OU and goniotomy OS with well-controlled IOP on multiple eye drops
- Monocular

Plan:
- Severity of vision loss and visual field loss seems out of proportion to degree of cupping described; assess for pallor of the optic nerve OU and check color vision; if non-glaucomatous etiology suspected then refer to neuro-ophthalmology.
- Assess tolerability of and adherence to current eye drop regimen.
- Assess stability of VF and OCT.
- Refer for low vision evaluation.
- Given monocular status, follow-up in 2 months.
- Ensure use of polycarbonate lenses.
- Advise patient regarding "return precautions" (e.g., redness, pain, decreased vision, light sensitivity).
- Counsel patient regarding irreversibility of glaucomatous optic neuropathy and need for vigilant follow-up.

Supplementary Figure 2: The figure juxtaposes the responses generated by ChatGPT and eight glaucoma specialists to a randomly selected patient from our cases. The left panel illustrates the dual-prong prompt provided to GPT-4, leading to rated AI-generated response. The right panel reflects responses from the specialists. This specific GPT-4 answer exhibited superior performance, with higher average accuracy (mean: 8.29, median: 9, std: 1.25) and completeness (mean: 5.14, median: 5, std: 0.69) than the specialists' accuracy (mean: 7.38, median: 8, std: 2.25) and completeness (mean: 4.22, median: 5, std: 1.40). Notably, the 5th specialist response achieved the highest accuracy (mean: 9.33, median: 10.00, std: 1.03) and completeness (mean: 5.33, median: 5.50, std: 0.82), while the 3rd specialist response represented the lowest average accuracy (mean: 4.67, median: 5.00, std: 2.42) and completeness (mean: 2.83, median: 2.50, std: 1.17).

© 2024 Huang AS et al. *JAMA Ophthalmology*.

eFigure 3. GPT vs Specialist in Glaucoma and Retina Clinical Scenarios

A: GPT vs Specialist in Glaucoma Clinical Scenarios

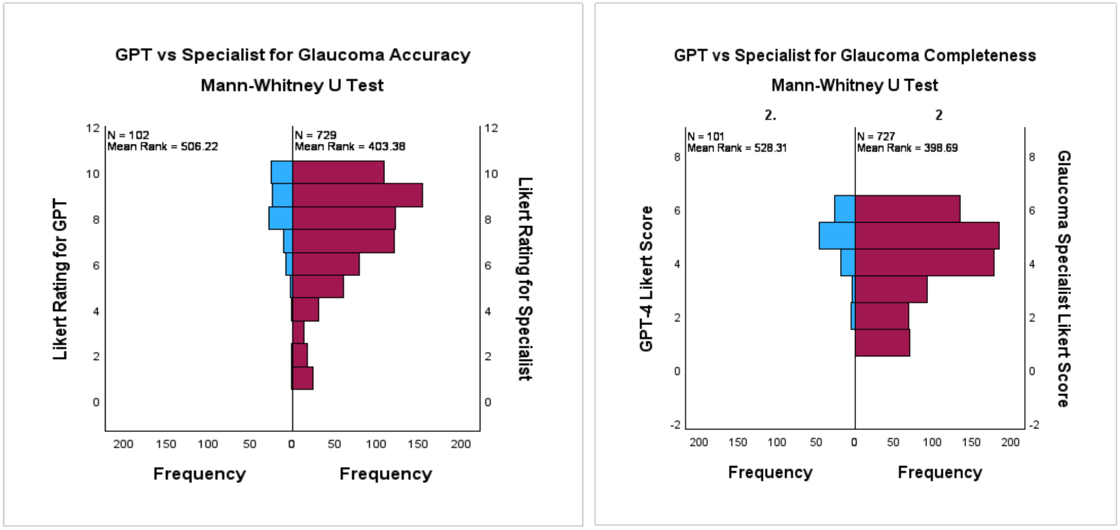

B: GPT vs Specialist in Retina Clinical Scenarios

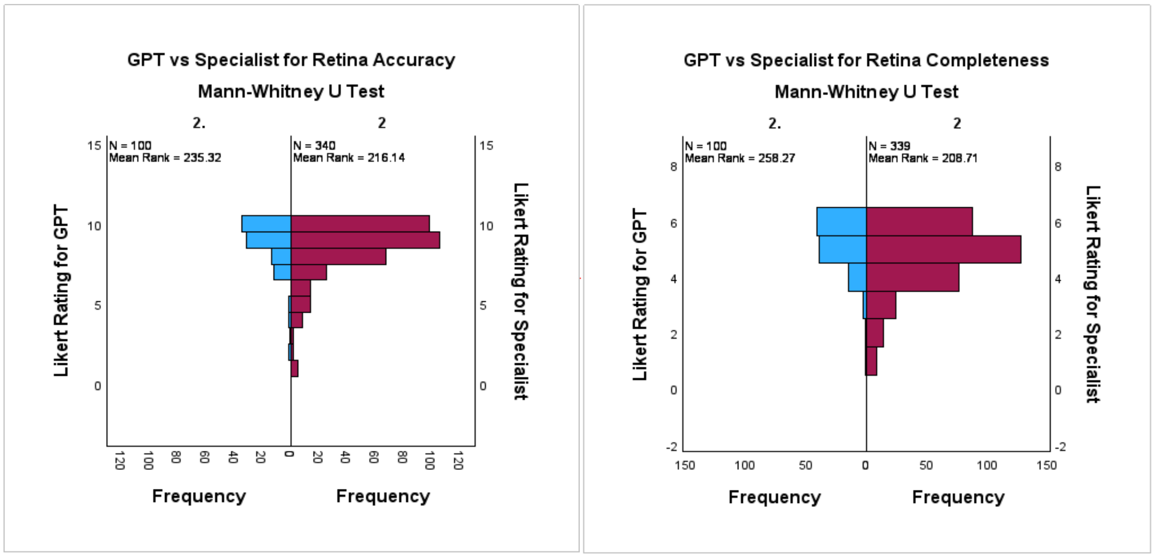

Supplementary Figure 3: Panels 3A and 3B present the results of a blinded evaluation comparing GPT-4 and specialists in glaucoma and retina clinical scenarios (combination of questions and cases), respectively. Each chart, derived from the Mann-Whitney U test, displays mean rank frequencies with the Likert scale ratings on the Y-axis, indicating perceived accuracy and completeness. Evaluations were conducted by several undisclosed graders, all masked to the respondents' identity and barred from assessing their responses. Fifteen total graders were involved.

Interpretation of U statistic: The Mann-Whitney U test is a non-parametric test used to compare differences between two independent groups when the dependent variable is either ordinal or continuous, but not normally distributed. This test is particularly suitable for our data set as it does not assume a normal distribution of the response scores and is robust against outliers, making it ideal for evaluating the Likert scale-based assessments. By using the U statistic, we were able to effectively measure and compare the central tendencies of the two groups' responses.

© 2024 Huang AS et al. *JAMA Ophthalmology*.

eAppendix. Glaucoma and Retina Ophthalmology Patient Cases

Glaucoma Patient Cases:

1. **One liner:** 55 y/o M h/o BRVO OD with NVE s/p sectoral PRP (2017), ocular hypertension, cupping OU, +fam h/o glaucoma. Meds: xal hs/hs, Brinzolamide 2/2.
Exam: VA 20/20 OU, IOP 15/13, Tmax: 22/24, PERRL no rAPD. **Slit lamp**: LL-normal OU. Conj/sclera-white and quiet OU. Cornea-clear OU. Anterior chamber: deep and quiet OU. Iris: round and reactive OU. Lens: NS 1+ OU. 7. CCT: 578/557. Gonio: grade 4 to CBB OU. **Dilated fundus exam**: Disc: 0.7/0.6 C:D OU, no edema or pallor, Macula: flat OU. Vessels: old BRVO OD superotemporal, normal OS. Periphery: as above. **Perimetry**: OD Inferior arcuate corresponding to BRVO, OS full. **RNFL:** 78/94, with superior thinning OD.

2. **One liner:** 80 y/o F h/o NTG and ERM OD. Gtts: cosopt 2/2, brim 2/2, xal hs/hs
Exam: VA: 20/25 OD, 20/20 OS. IOP 10/12, Tmax 18/18. PERRL, no rAPD. **Slit lamp**: LL: normal OU. Conj/sclera: white and quiet OU. Cornea: clear OU. Anterior chamber: deep and quiet OU. Iris: round and reactive OU. Lens: PCIOL OU. CCT: 571/602. Gonio: grade 4 CBB OU. **Dilated fundus exam**: Disc: 0.8 OU, +Peripapillary atrophy OU. Macula: flat OU. Vessels: normal OU. Periphery: normal OU. **Perimetry**: OD full | OS superior arcuate. **RNFL:** 91/79, thinning inferiorly OS.

3. **One liner:** 63 y/o F h/o SLE, uveitic glaucoma OU, CEIOL OU, left-sided CVA (right-sided weakness). Gtts: cos 2/2, xal 0/hs. **Exam:** VA 20/20 OU. IOP 12/11, Tmax: 35 OU. PERRL, no rAPD. **Slit lamp:** LL: normal OU. Conj/sclera: white and quiet OD, 1+ injection OS. Cornea: clear OU. Anterior chamber: deep and quiet OU. Iris: round and reactive OU, atrophy OD. Lens: PCIOL OU. Anterior vitreous: normal OU. Gonio: grade 4 CBB OU. **Dilated fundus exam**: Disc: 0.4/0.6. Macula: normal, flat OU. Vessels: normal OU. Periphery: normal OU. **Perimetry**: Right inferior quadrantanopia OU, OS superior arcuate. **RNFL:** 87/72, full OD, inferior thinning OS.

4. **One liner:** 75 y/o F with h/o HTN, NIDDM, NTG, no fam h/o glaucoma, gtts: simbrinza 3/3, xal hs/hs. **Exam:** VA OD 20/30, OS 20/20. IOP 13/12, Tmax 16/17. PERRL, no rAPD OU. **Slit lamp:** LL: normal OU. Conj/sclera: white and quiet OU. Cornea: clear OU. Anterior chamber: deep and quiet OU. Iris: round OU. Lens: NS 1+ OU. CCT: 492/514. Gonio: grade 4 CBB OU. **Dilated fundus exam**: Disc: 0.5 OU C:D OU, no edema or pallor. Macula: norma, flat OU. Vessels: normal OU. Periphery: normal OU. **Perimetry**: Grossly full, poor reliability, cloverleaf **RNFL:** 72/72, OD full, OS inferior thinning.

5. **One liner:** 72 y/o F h/o ANA s/p LI OU with pre-perimetric changes; +fam h/o glaucoma; gtts: xal hs/hs. **Exam**: VA 20/25 OU. IOP 18/16. PERRL, no rAPD OU. **Slit lamp:** LL: normal OU. Conj/sclera: tr injection OU. Cornea: PEE OU. AC: deep and quiet. Iris: round, patent LI OU. Lens: NS 2+ OU. CCT: 550/535. Gonio: grade 2, posterior trabecular meshwork OU. **Dilated fundus exam**: Disc: 0.85/0.6, no edema or pallor. Macula: normal, flat OU. Vessels: normal OU. Periphery: normal OU. **Perimetry**: Full OU. **RNFL:** 72/70; OD superior and inferior thinning, OS inferior thinning.

© 2024 Huang AS et al. *JAMA Ophthalmology*.

6. **One liner:** 74 y/o F h/o ANA complicated by angle closure OU s/p LI OU; gtts: Xalatan hs/hs, no fam h/o glaucoma.
Exam: VA 20/25 OU. IOP 9/12; Tmax unknown 18 on gtts. PERRL, no rAPD OU. **Slit lamp**: LL: normal OU. Conj/sclera: white and quiet. Cornea: PEE OU. AC: narrow. Iris: round, patent LI OU. Lens: NS 1+ OU. CCT: not documented. Gonio: Grade 1 OU, opens to scleral spur OD, posterior trab mesh OS. **Dilated fundus exam**: Disc: 0.8/0.7, no edema or pallor. Macula: normal, flat OU. Vessels: normal OU. Periphery: normal OU. **Perimetry**: Nasal step and superior arcuate OS>OD. **RNFL**: OD 63 (I thin) OD/ OS 49 (S/I/N thinning).

7. This case was removed due to inconsistency with H&P descriptions.

8. **One liner:** 73 y/o M h/o HTN, CEIOL OU, POAG h/o trabeculectomy OU; gtts: Xalatan hs/hs.
Exam: VA OD 20/40 | OS 20/125. IOP 8/8; Tmax unknown. +APD OS. **Slit lamp**: LL: normal OU. Conj/sclera: white and quiet, bleb superiorly OU. Cornea: clear OU. AC: deep and quiet. Iris: Superior surgical iridectomy OU. Lens: PCIOL OU. CCT: 475/483. Gonio: scattered PAS. **Dilated fundus exam**: Disc: 0.8/0.9; pallor OS. Macula: normal, flat OU. Vessels: normal OU. Periphery: normal OU. **Perimetry**: OD full | OS central island. **RNFL**: 60/58.

9. **One liner:** 78 y/o M h/o CACG s/p LI OU, CEIOL OU s/p Kahook goniotomy OS; gtts: Alphagan 2/2, Cosopt 2/2, Xalatan hs/hs, Rhopressa hs/hs.
Exam: VA OD 20/100; OS HM. IOP 7/7; Tmax 23/23. PERRL, no rAPD OU. **Slit lamp**: LL: normal OU. Conj/sclera: white and quiet. Cornea: PEE OU. AC: deep and quiet OU. Iris: patent LI OU. Lens: PCIOL OU. CCT: 529/527. Gonio: grade 3 OU, posterior trab mesh-scleral spur OU. **Dilated fundus exam**: Disc: 0.7 OU. Macula: normal, flat OU. Vessels: normal OU. Periphery: normal OU. **Perimetry**: Inferotemporal islands OU. **RNFL**: 61/61, thinning OU.

10. **One liner:** 57 y/o M h/o CEIOL OS with Kahook goniotomy OS, SLT OU, trab OU; gtts: Alphagan 2/2.
Exam: VA OD 20/125 (ph 20/25); OS 20/25. IOP: 20/24; Tmax 30/38. PERRL, no rAPD OU. **Slit lamp**: LL: normal OU. Conj/sclera: white and quiet, bleb superiorly OU, flat. Cornea: PEE OU. AC: deep and quiet. Iris: superior surgical iridectomy OU. Lens: OD cortical and NS | OS PCIOL. CCT: 588/597. Gonio: Grade 4 OU to CBB. **Dilated fundus exam**: Disc: 0.4/0.7. Macula: normal, flat OU. Vessels: normal OU. Periphery: normal OU. **Perimetry**: OD full | OS inferior arcuate. **RNFL**: 68/58, OD inferior thinning; OS superior thinning.

Retina Patient Cases:

1. **One liner:** 40F with history of idiopathic ESRD (on PD), hypertension, myopia presents with complaint of black curtain over nasal visual field of the right eye for 1 month. Denies flashes, floaters, progression of visual change over the last month, trauma, personal or family history of retinal disease.
Exam: VA 20/20 OU. IOP 14/15. PERRL no rAPD. Colors 12/12 ishihara color plates OU. **Slit lamp**:

© 2024 Huang AS et al. *JAMA Ophthalmology*.

LL: normal OU. Conj/sclera: white and quiet OU. Cornea: clear OU. Anterior chamber: deep and quiet OU. Iris: round and reactive OU. Lens: clear OU. Anterior vitreous: Schafer negative OU. **Dilated fundus exam**: Disc: 0.3 C:D OU, tilted OS; no edema or pallor. Macula: normal, flat OU. Vessels: normal OU. Periphery: macula on RRD from 11 to 7 o clock, horseshoe tear 10 o clock.

2. **One liner:** 26M with no medical or ocular history presents for evaluation of blurring of superior visual field of the left eye for 2 weeks. Denies flashes, floaters, history of trauma or myopia. States that prior to onset of visual symptom was using ice chipper and thought something may have went into his eye, but he went to the emergency department who did not find any abnormal exam findings.
   Exam: VA: 20/20 OD, 20/200 OS no improvement with pinhole. IOP 10/12. PERRL, rAPD present on the left. Confrontational visual fields: full OD, superior hemifield relative scotoma. Colors: 12/12 Ishihara color plates OU. **Slit lamp**: LL: normal OU. conj/sclera: white and quiet OU. Cornea: clear OU. Anterior chamber: deep and quiet OU. Iris: round and reactive OU. Lens: clear OU. Anterior vitreous: schafer sign positive OS, negative OD. **Dilated fundus exam**: Disc: 0.3 C:D OU, no edema or pallor. Macula: flat OD, macula elevated OS. Vessels: normal OU. Periphery: chorioretinal scars inferiorly OD; inferior rhegmatogenous retinal detachment 3 o clock to 8 o clock with 3 breaks between 4 and 5 o clock in the far periphery; hyaloid face detached inferiorly.

3. **One liner:** 33M with history of prediabetes presents with acute onset left flank pain for 12 hours, new floaters, pain, and significantly blurry vision in the left eye for the same period of time. Denies any history of similar episodes, flashes of light, eye surgery, ocular trauma, recent travel. **Exam:** VA 20/20 OD, 20/25 OS. IOP 16/11. PERRL, no rAPD. **Slit lamp:** LL: normal OU. Conj/sclera: white and quiet OD, 1+ injection OS. Cornea: clear OU. Anterior chamber: deep and quiet OD; 2mm hypopyon OS. Iris: round and reactive OU. Lens: clear OU. Anterior vitreous: normal OD, haze OS. **Dilated fundus exam**: Disc: 0.3 C:D OU, no edema or pallor. Macula: normal, flat OD; hazy view - grossly flat OS. Vessels: normal OD; poor view OS. Periphery: superotemporal extramacular subretinal white collection of fluid. **B scan:** flat OD; superotemporal fluid collection.

4. **One liner:** 33M with history of type-1 diabetes mellitus presents for diabetic eye exam. His most recent HgbA1c was 9.6 and has no visual complaints. **Exam:** VA 20/20 OU. IOP 18/19. PERRL, no rAPD OU. **Slit lamp:** LL: normal OU. conj/sclera: white and quiet OU. Cornea: clear OU. Anterior chamber: deep and quiet OU. Iris: round and reactive, no iris neovascularization OU. Lens: clear OU. Anterior vitreous: clear OU. **Dilated fundus exam**: Disc: 0.3 C:D OU, no edema or pallor. Macula: normal, flat OD; trace exudates OS. Vessels: normal OU. Periphery: dot-blot hemorrhages in 1 of four quadrants, extramacular exudates. **OCT**: no intra- or sub-retinal fluid OU, trace extramacular exudates OS. **Fluorescein angiography**: microaneurysms OU; no neovascularization everywhere or disc neovascularization; no capillary dropout.

5. **One liner:** 39M hx HTN, pre-diabetes, narrow angle glaucoma, dry eye presents with blurry vision of the right eye since this morning. Denies pain, foreign body sensation, discharge, trauma, nausea, vomiting. Does not use any glaucoma drops and has never had any procedure

© 2024 Huang AS et al. *JAMA Ophthalmology*.

on the eyes. **Exam:** VA 20/20 OU. IOP 12/12. PERRL, no rAPD OU. **Slit lamp:** LL: normal OU. conj/sclera: tr injection OU. Cornea: PEE OU. Anterior chamber: narrow OU, grade 1, occludable on gonioscopy. Iris: round and reactive, no iris neovascularization OU. Lens: clear OU. Anterior vitreous: clear OU. **Dilated fundus exam**: Dilated fundus exam deferred due to narrow angles. **Amsler grid testing:** Metamorphopsia present OD. Ishihara color plates: 12/12.

6. **One liner:** 30F with history of bilateral ICA ophthalmic artery aneurysm s/o angiogram and flow diversion 1 month ago presents with acute painless vision loss of the left eye. Woke upat 11AM this morning with only a small central island of her visual field preserved. Denies flashes, floaters, pain, headache, jaw claudication, fevers/chills, weight loss/weight gain, scalp tenderness. **Exam**: VA 20/20 OU. IOP soft to palpation OU. PERRL, rAPD present OS. Motility full and painless OU. **Slit lamp:** LL: normal OU. Conj/sclera: white and quiet OU. Cornea: clear OU. Anterior chamber: deep and quiet OU. Iris: round and reactive, no iris neovascularization OU. Lens: clear OU. Anterior vitreous: clear OU. **Dilated fundus exam**: Disc: 0.3 C:D OU, no edema or pallor. Macula: flat. Vessels: normal OU. Periphery: diffuse retinal whitening with preservation of macula and area between optic nerve and macula.

7. **One liner:** 22M no past medical or ocular history presents with difficulty focusing at near for 1 week. Uses reading glasses. Denies headache, dizziness, diplopia, blurry vision, recreational drug use. Takes adderall for ADHD. **Exam**: VA 20/20 OU. IOP 16/17. PERRL, no rAPD OU. Motility full and painless. **Slit lamp:** LL: normal OU. conj/sclera: white and quiet OU. Cornea: clear OU. Anterior chamber: deep and quiet OU. Iris: round and reactive, no iris neovascularization OU. Lens: clear OU. Anterior vitreous: clear OU. **Dilated fundus exam**: Disc: 0.3 C:D OU, no edema or pallor. Macula: white parafoveal lesion OU (OS>OD). Vessels: normal OU. Periphery: normal OU. **OCT macula**: outer retinal hyperreflective lesion with RPE disruption OU. **Fundus autofluorescence**: focal parafoveal hypoautofluorescence OU. **Fluorescein angiography**: normal OU.

8. **One liner:** 86F history of hypothyroidism, glaucoma presents with painless vision loss that occurred possibly 9 days ago although patient not entirely sure of time course, unclear whether it was sudden or gradual. Endorses mild left sided headaches. Denies scalp tenderness, jaw claudication, myalgias, weight loss, fever, diplopia. **Exam**: VA 20/25 OD NLP OS. IOP 18/29. Round/reactive OD, no reaction OS, +rAPD OS. Motility full and painless. **Slit lamp:** LL: normal OU. conj/sclera: white and quiet OU. Cornea: clear OU. Anterior chamber: deep and quiet OU. Iris: round and reactive, no iris neovascularization OU. Lens: Nuclear and cortical changes OU. Anterior vitreous: clear OU. Gonioscopy: grade 3 without NVA OU. **Dilated fundus exam**: Disc: 0.9 C:D OU; superonasal disc hemorrhage OS. Macula: normal OD; scattered DBH OS. Vessels: attenuated OU. Periphery: normal OD; diffuse dot blot hemorrhages OS.

9. **One liner:** 75F hx of RA (previously on Rituxan infusions) presents with acute painless vision loss OD. Denies trauma, flashes, floaters, pain. Takes ASA 81mg for unknown reason. **Exam**: VA HM OD, 20/20 OS. IOP 13/13. PERRL, no rAPD OU. Motility full and painless. **Slit lamp:** LL: normal OU. conj/sclera: white and quiet OU. Cornea: clear OU. Anterior chamber: 1mm hyphema OD; 4+RBC OD; deep/quiet OU. Iris: round, reactive OU. No NVI OU. Lens: PCIOL, phacodonesis OU;

© 2024 Huang AS et al. *JAMA Ophthalmology*.

blood between PCIOL and posterior lens capsule, blocking inferior 50% of lens OD. Anterior vitreous: clear OU. **Dilated fundus exam**: No view OD. OS with PVD and peripapillary atrophy, 0.3 C:D. **B scan**: flat, no heme/RD/mass OU.

10. **One liner:** 25M with no past medical or ocular history presents with evaluation of blurry vision OU. Was prescribed glasses by outside optometrist which did not help)**. Exam**: VA 20/60 OU; ph 20/50 OD (did not improve with refraction). IOP 7/11. PERRL, no rAPD OU. Motility full and painless. **Slit lamp:** LL: normal OU. conj/sclera: white and quiet OU. Cornea: clear OU. Anterior chamber: deep and quiet OU. Iris: round and reactive, no iris neovascularization OU. Lens: clear OU. Anterior vitreous: clear OU. **Dilated fundus exam**: Disc: 0.2 C:D OU, no edema or pallor. Macula: atrophy and mottling OU. Vessels: normal OU. Periphery: posterior pole pigmentary changes.

© 2024 Huang AS et al. *JAMA Ophthalmology*.
