# Supplementary material for: Assessment of a Large Language Model’s Responses to Questions and Cases About Glaucoma and Retina Management
Source: JAMA Ophthalmol. 2024 Feb 22;142(4):371–5. doi: 10.1001/jamaophthalmol.2023.6917 (PMC10884943; doi:10.1001/jamaophthalmol.2023.6917)
Supplement: Supplement 2. — Data Sharing Statement [file jamaophthalmol-e236917-s002.pdf]

Data Sharing Statement

Huang. Assessment of a Large Language Model's Responses to Questions and Cases About Glaucoma and Retina Management. *JAMA Ophthalmol*. Published February 22, 2024. doi:10.1001/jamaophthalmol.2023.6917

Data
Data available: Yes
Data types: Deidentified participant data
How to access data: andyshihuang@Gmail.com
When available: With publication

Supporting Documents
Document types: None

Additional Information
Who can access the data: Researchers whose proposed use of the data has been approved
Types of analyses: For research on AI within Mount Sinai Health System
Mechanisms of data availability: With a signed data access agreement.
